# The relationships between physical activity, lumbar multifidus muscle morphology, and low back pain from childhood to early adulthood: a 12-year longitudinal study

**DOI:** 10.1038/s41598-022-12674-8

**Published:** 2022-05-25

**Authors:** Erin Cunningham, Niels Wedderkopp, Per Kjaer, Amber Beynon, Jeremy Noble, Jeffrey J. Hebert

**Affiliations:** 1 Faculty of Kinesiology, University of New Brunswick, Fredericton, Canada; 2 Department of Sports Science and Clinical Biomechanics, University of Southern Denmark, Odense, Denmark; 3 Health Sciences Research Centre, UCL University College, Odense, Denmark; 4 College of Science, Health, Engineering and Education, Murdoch University, Murdoch, Australia; 5 Department of Chiropractic, Faculty of Medicine, Health and Human Sciences, Macquarie University, Sydney, Australia; 6 The Research Unit of Pediatrics, Department of Clinical Research, The Faculty of Health Sciences, University of Southern Denmark, Odense, Denmark

**Keywords:** Anatomy, Health care

## Abstract

We investigated the longitudinal associations between physical activity (PA), lumbar multifidus morphology, and impactful low back pain (LBP) in young people. Nine-year-old children were recruited from 25 primary schools and followed up at age 13, 16, and 21 years. We measured PA with accelerometers at age 9, 13, and 16; quantified patterns of lumbar multifidus intramuscular adipose tissue (IMAT) change from 13 to 16 years using magnetic resonance imaging; and recorded LBP and its impact with standardised questionnaires and interviews. Associations were examined with crude and adjusted logistic or multinomial models and reported with odds ratios (OR) or relative risk ratios (RRR). We included data from 364 children (mean[SD] age = 9.7[.4] years). PA behaviour was not associated with LBP. Having persistently high IMAT levels at age 13 and 16 was associated with greater odds of LBP (OR[95% CI] = 2.98[1.17 to 7.58]). Increased time in moderate and vigorous intensity PA was associated with a lower risk of higher IMAT patterns (RRR[95% CI] = .67[.46 to .96] to .74[.55 to 1.00]). All associations became non-significant after adjusting for sex and body mass index (BMI). Future studies investigating the relationships between PA behaviour, lumbar multifidus IMAT, and impactful LBP should account for potential confounding by sex and BMI.

## Introduction

Back pain is the leading cause of disability worldwide^1^. The socioeconomic burden of low back pain is substantial; from 2012 to 2014, the average annual back pain related costs in the United States was USD$315 billion^2^. For some, low back pain starts in childhood and becomes more common in adolescence and adulthood^3,4^. The prevalence of back pain in youth ranges from 9 to 61%, and tracks from childhood to adulthood^5,6^. Little is known about the cause of low back pain in young people.

Physical activity behaviour may be associated with low back pain. Greater levels of physical activity are reportedly associated with back pain^7^. Moreover, activity intensity may play an important role; vigorous activity is associated with increased risk of low back pain, whereas moderate activity shows a protective association with back pain^8^.

Several studies have identified relationships between back muscle morphology and back pain. Greater functional cross-sectional area of the lumbar multifidus and erector spinae muscles are associated with less risk of lower back pain^9^, while smaller size and fatty infiltration of the lumbar multifidus muscles increased the likelihood of back pain^10^.

Studies examining the relationships between low back pain and muscle morphology, as well as low back pain and physical activity, have focused on adult populations^4,7–11^. Moreover, there has been a lack of research considering the potential impact of physical activity together with muscle morphology.

The aim of this study was to investigate the longitudinal relationships between physical activity, lumbar multifidus morphology, and low back pain in young people. Study objective one was to investigate the associations between physical activity behaviour and low back pain. Objective two was to investigate the associations between lumbar multifidus intramuscular adipose tissue (IMAT) and low back pain. Objective three was to explore the associations between physical activity behaviour and lumbar multifidus IMAT.

We hypothesised that (i) moderate intensity physical activity would be associated with a decreased likelihood of low back pain while vigorous intensity activity would be associated with an increased likelihood; (ii) higher IMAT would be associated with a greater likelihood of low back pain; and (iii) physical activity would be negatively associated with IMAT.

## Results

In total, data from 364 children were included for analysis. The number of participants included in the models ranged from 179 to 308 depending on the time points and variables of interest. Descriptive demographic data at all time points are reported in Table 1.

**Table 1:** Descriptive baseline and follow-up demographic information^a^.

Variables	Age 9 (N = 277)	Age 13 (N = 315)	Age 16 (N = 363)	Age 21 (N = 243)
Age (y)	9.7 ± .4	13.1 ± .3	15.7 ± .4	21.8 ± .4
Female sex; n (%)	151 (54.5%)	172 (54.6%)	203 (55.9%)	134 (55.1%)
Body Mass Index (kg/m^2^)	17.1 ± 2.2	19.4 ± 2.6	21.1 ± 3.0	23.8 ± 3.7

^a^All values are reported as mean ± standard deviation.

### Physical activity and low back pain

Crude and sex-, BMI-, and accelerometer wear time-adjusted analyses showed no associations between physical activity behaviour at age 16 and impactful LBP at age 21 (Table 2).

**Table 2:** Adjusted and unadjusted odds ratios for physical activity at age 16 and 21 years and low back pain at age 21 years^a^.

	LBP age 21(crude)	LBP age 21(adjusted)
Physical activity age 16	N = 194	N = 194
Sedentary	.97 (.93 to 1.01)	.97 (.92 to 1.02)
Light	1.01 (.97 to 1.06)	1.01 (.96 to 1.06)
Moderate	1.13 (.90 to 1.42)	1.21 (.95 to 1.55)
Vigorous	1.00 (.75 to 1.35)	1.08 (.79 to 1.48)

^a^All values are odds ratios (95% confidence interval) per 10 min of physical activity.

All estimates are adjusted for accelerometer wear time. Adjusted models additionally control for sex and body mass index at age 21.

### Muscle morphology and low back pain

Mean IMAT measurements at ages 13 and 16, stratified by patterns of change, are reported in Table 3. Participants with persistently high levels of lumbar multifidus IMAT from age 13 to 16 had increased odds of impactful LBP at age 16 (OR [95% CI] = 2.98 [1.17–7.58]) compared to children with persistently low IMAT. However, this association became non-significant after adjusting for sex and BMI (OR [95% CI] = 2.14 [0.81–5.63]). There were no other associations between IMAT patterns and impactful LBP at age 16 or 21 years (Table 4).

**Table 3:** Mean proportion of intramuscular adipose tissue of the lumbar multifidus muscle^a^.

IMAT pattern of change	IMAT age 13	IMAT age 16
	N = 308	N = 308
Persistent Low	.10 ± .02	.10 ± .02
Decreasing IMAT	.21 ± .04	.14 ± .04
Persistent Mid IMAT	.17 ± .02	.17 ± .02
Increasing IMAT	.15 ± .03	.22 ± .05
Persistent High IMAT	.27 ± .06	.27 ± .06

^a^All values are reported as mean ± standard deviation of the maximal proportion of intramuscular adipose tissue (IMAT).

**Table 4:** Crude and adjusted odds ratios for patterns of change in lumbar multifidus intramuscular fat from age 13 to 16 and low back pain at age 16 and 21^a^.

	LBP age 16 (crude)	LBP age 16 (adjusted)	LBP age 21 (crude)	LBP age 21 (adjusted)
Participants	N = 308	N = 308	N = 182	N = 182
Persistent Low IMAT	reference	reference	reference	reference
Decreasing IMAT	1.70 (.66 to 4.41)	1.36 (.52 to 3.60)	.74 (.29 to 1.89)	.60 (.23 to 1.60)
Persistent Mid IMAT	1.56 (.52 to 4.65)	1.24 (.39 to 3.92)	.68 (.22 to 2.07)	.53 (.16 to 1.70)
Increasing IMAT	1.80 (.67 to 4.81)	1.33 (.49 to 3.78)	.93 (.35 to 2.48)	.67 (.23 to 1.93)
Persistent High IMAT	**2.98 (1.17 to 7.58)**	2.14 (.81 to 5.63)	.91 (.35 to 2.35)	.63 (.22 to 1.82)

^a^All values are odds ratios (95% confidence interval). Adjusted models included sex and BMI at age 21. Intramuscular adipose tissue is abbreviated as IMAT. Persistent low IMAT was used as a reference category.

Significant values are in [bold].

### Physical activity and muscle morphology

At age 9, increased time in moderate intensity activity was associated with a lower risk of persistent moderate IMAT, compared to low IMAT levels from 13 and 16 years (RRR [95% CI] = 0.71 [0.53–0.95]). Similarly, increased time in moderate intensity activity at age 16 was associated with decreased risk of persistent moderate (RRR [95% CI] = 0.70 (0.51–0.97]), increasing (RRR [95% CI] = 0.74 [0.55–1.00]), and persistent high (RRR [95% CI] = 0.72 [0.54–0.97]) IMAT patterns. Greater vigorous physical activity at age 16 was associated with lower risk of increasing IMAT (RRR [95% CI] = 0.67 [0.46–0.96]) compared to the low IMAT pattern from 13 to 16 years. However, all associations became non-significant after adjusting for sex and BMI (Table 5).

**Table 5:** Adjusted and unadjusted associations between physical activity at ages 9 and 16 and muscle morphology changes between ages 13 and 16.

		Persistent Low IMAT	Decreasing IMAT	Persistent moderate IMAT	Increasing IMAT	Persistent high IMAT
**Physical activity at age 9 (N = 179)**						
Sedentary	Crude	reference	1.04 (.96 to 1.11)	1.04 (.97 to 1.13)	1.05 (.97 to 1.13)	1.04 (.97 to 1.11)
	Adjusted	reference	1.05 (.89 to .13)	1.05 (.97 to 1.14)	1.07 (.98 to 1.16)	1.06 (.98 to 1.14)
Light	Crude	reference	1.01 (.91 to 1.11)	1.03 (.92 to 1.16)	1.00 (.91 to 1.10)	1.03 (.94 to 1.12)
	Adjusted	reference	.99 (.89 to 1.10)	1.01 (.98 to 1.14)	.96 (.87 to 1.07)	.99 (.90 to 1.10)
Moderate	Crude	reference	.83 (.67 to 1.03)	**.71 (.53 to .95)**	.78 (.57 to 1.06)	.83 (.67 to 1.03)
	Adjusted	reference	.86 (.69 to 1.08)	.77 (.57 to 1.04)	.91 (.65 to 1.27)	.98 (.77 to 1.26)
Vigorous	Crude	reference	.91 (.68 to 1.23)	.81 (.57 to 1.15)	.76 (.51 to 1.13)	.73 (.53 to 1.02)
	Adjusted	reference	.96 (.71 to 1.30)	.91 (.64 to 1.04)	.90 (.58 to 1.40)	.88 (.60 to 1.26)
**Physical activity at age 16 (N = 234)**						
Sedentary	Crude	reference	1.01 (.95 to 1.23)	1.02 (.96 to 1.09)	1.02 (.96 to 1.07)	1.04 (.99 to 1.10)
	Adjusted	reference	1.00 (.95 to 1.05)	1.01 (.95 to 1.07)	1.00 (.95 to 1.06)	1.02 (.97 to 1.08)
Light	Crude	reference	1.00 (.95 to 1.06)	1.00 (.93 to 1.07)	.99 (.93 to 1.05)	.96 (.91 to 1.02)
	Adjusted	reference	1.01 (.96 to 1.06)	1.01 (.94 to 1.07)	1.00 (.93 to 1.06)	.98 (.92 to 1.04)
Moderate	Crude	reference	.91 (.68 to 1.06)	**.70 (.51 to .97)**	**.74 (.55 to 1.00)**	**.72 (.54 to .97)**
	Adjusted	reference	1.01 (.75 to 1.38)	.78 (.55 to 1.11)	.90 (.66 to 1.23)	.93 (.70 to 1.24)
Vigorous	Crude	reference	.96 (.68 to 1.34)	.78 (.51 to 1.21)	**.67 (.46 to .96)**	.69 (.47 to 1.00)
	Adjusted	reference	1.12 (.78 to 1.61)	.94 (.60 to 1.46)	.86 (.57 to 1.29)	.93 (.64 to 1.38)

^a^All values are relative risk ratios (95% confidence interval) per 10 min of physical activity. All estimates adjusted for accelerometer wear time. Adjusted values additionally control for sex and body mass index at age 21.

Intramuscular adipose tissue is abbreviated as IMAT.

Significant values are in [bold].

## Discussion

This study aimed to investigate the longitudinal relationships between physical activity, lumbar multifidus morphology, and impactful low back pain in young people. We found that persistently high levels of lumbar multifidus IMAT from age 13 to 16 were associated with an increased likelihood of impactful low back pain at age 16. We found no relationships between physical activity behaviour at age 16 and likelihood of impactful low back pain at age 21. Increased time in moderate intensity activity was associated with decreased risk of moderate, high, and increasing patterns of lumbar multifidus IMAT compared to persistent low IMAT from age 13 to 16. Greater vigorous physical activity at age 16 was associated with decreased risk of an increasing IMAT pattern only. These estimates became non-significant after adjusting for sex and BMI. This means that the associations investigated in the current study may be confounded by sex, BMI, or both.

The results of previous studies investigating the relations between physical activity behaviour and spinal pain have produced conflicting results. Cross-sectional analyses have reported no associations between self-reported physical activity and impactful low back pain^7^ and between device-measured physical activity and low back pain intensity^12^ in young people. Conversely, a prospective study found that high levels of physical activity in childhood protected against back pain in adolescence^13^. Another prospective study of children and adolescents reported that the relationship between physical activity and spinal pain in children may depend on activity intensity; moderate activity was associated with a decreased likelihood and vigorous activity was associated with an increased likelihood of future spinal pain^8^. In contrast, we found no evidence that the nature of the relationship between physical activity and spinal pain was modified by activity intensity. A point of difference between the current study and previous investigations is that we controlled for BMI, while the previous studies did not account for this potential source of confounding. Moreover, differences between the measures of spinal pain may have played a role; the only other study to distinguish between moderate and vigorous intensity activity^8^ measured spinal pain frequency within one-week sampling windows, while the current study measured low back pain occurrence over long-term intervals.

Evidence from adult studies suggests that increased IMAT in the lumbar multifidus is associated with an increased likelihood of low back pain^9,14^. Although we identified evidence of a similar relationship in our univariate analysis, this association appeared to be confounded by sex and BMI—covariates not totally accounted for in adult studies. Alternatively, comparisons between youth and adult populations may be complicated by differences in the nature of the relationship between lumbar multifidus IMAT and low back pain over the life course. For example, children^15^and adults^16^ experience different trajectories of spinal pain, and lumbar multifidus IMAT appears to increase as a function of age^11,17^.

While it may seem logical that physical activity would relate to spinal muscle morphology, we found no clear evidence for such a relationship. We are unaware of previous studies reporting links between health-related physical activity and lumber multifidus morphology. However, there is evidence that extreme sedentary behaviour may result in lumbar multifidus atrophy. For example, spaceflight^18,19^ and prolonged head-down tilt bed rest^20^ may reduce lumbar multifidus size and increase IMAT; changes that are potentially reversible with exercise training^21^. Our results do accord with studies reporting little to no relations between lumbar multifidus IMAT and lumbar multifidus function^22^, prognostic factors associated with exercise outcomes for patients with low back pain^23^, as well as lumbar multifidus size and tests of physical performance^24^.

Currently, the antecedents of lumbar multifidus IMAT infiltration remain poorly understood and this will be an important topic for future study. Preliminary evidence suggests that lumbar multifidus degeneration may result from mechanical pathology^25^ or degeneration-induced inflammatory changes of the muscle^26^. A better understanding of the mechanisms responsible for muscle degeneration may help to guide future research efforts.

### Strengths and limitations

Strengths of the current study included the prospective design with repeated measures of physical activity, low back pain, and muscle morphology obtained over 11 years using robust methods. The use of accelerometry resulted in objective physical activity measures which likely reduced recall and social desirability bias, compared to alternate approaches such as self-reported activity. Further, the back pain measure included an estimate of impact and we therefore limited the potential influence of more trivial pain episodes. Finally, we used reliable MRI methods to quantify the amount of IMAT in the lower lumbar multifidus muscles.

However, the study results should also be considered in light of several study limitations. Accelerometry is unable to capture all modes of physical activity such as swimming and cycling and therefore, some sources of activity were not captured by our approach. Although we measured low back pain outcomes with standard measures and considered the impact of pain on care-seeking and daily activities, we did not consider other clinically relevant aspects of pain such as intensity, frequency, and duration. Despite controlling for the variance associated with sex and BMI, these issues represent potential sources of residual confounding in our analyses.

## Conclusion

In this sample of young people, having a persistently high level of lumbar multifidus IMAT was associated with an increased likelihood of reporting impactful low back pain. Although physical activity behaviour was not associated with low back pain, it was associated with different patterns of lumbar multifidus IMAT. However, all associations became non-significant in the adjusted models, indicating that these relationships may be confounded by sex and BMI. These results support a complex mechanism for the development of low back pain in youth that is not fully explained by physical activity behaviours and lumbar multifidus muscle morphology. Future studies investigating the interrelationships between physical activity, muscle morphology, and back pain should, at minimum, account for potential confounding by sex and BMI.

## Methods

### Study design and participants

We analyzed physical activity, muscle morphology, and back pain data from the Danish cohort of the European Youth Heart Study, a longitudinal investigation of cardiovascular disease and diabetes risk factors in young people. A two-stage cluster sample was used to randomly select a representative sample of third-grade students attending one of 25 public primary schools. The primary sampling units were schools and the secondary sampling units were students. Schools were stratified by socio-economic status, geography (urban, suburban, rural), and the age and sex of enrolled students and weighted according to the size of the school^27^. A random sample of third-grade students enrolled in the schools were then selected for inclusion. Children with serious, chronic medical conditions that precluded their participation were excluded. Additional children meeting these selection criteria were enrolled in subsequent years.

**Figure 1:**
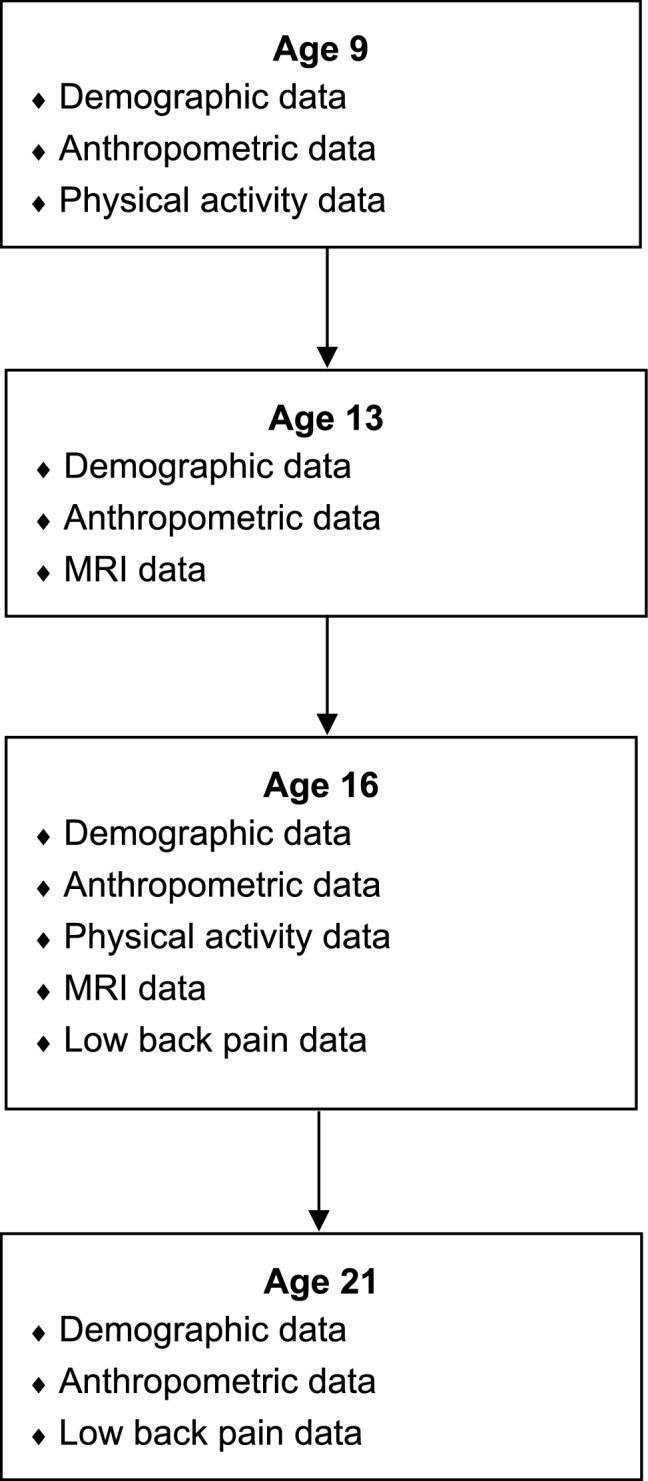
Data collection time-points for the physical activity, muscle morphology, and low-back pain data.

Physical activity behaviour was measured at 9, 16, and 21 years of age. Muscle morphology was measured at age 13 and 16 years. Back pain outcomes were measured at age 16 and 21 years (Fig. 1). The Regional Committees on Health Research Ethics for Southern Denmark (reference nos. 20000045, 96/272) and the University of New Brunswick (reference no. REB#2019–056) approved the study and use of the data was approved by the Danish Data Protection Agency (reference no. 2000-5-3-0037). All parents provided written informed consented in regard to study participation prior to enrolment and children provided verbal consent at the time of testing, if any child declined then they did not participate. All relevant guidelines and regulations were followed surrounding scientific research and human participants.

### Exposure and outcome variables

#### Anthropometry

Standard measures of height and weight were obtained using a SV-Seca 710 stadiometer and beam scale weight (Seca Precision for Health, Hamburg, Germany)^28^. Body-mass index (BMI) was calculated as weight[kg]/(height[m])^2^.

#### Physical activity

Physical activity was measured using MTI 7164 accelerometers (Manufacturing Technology Incorporated, Shalimar, FL)^27^ during seven-day measurement periods^29^. A trained graduate research assistant with several years’ experience using accelerometry with children secured the accelerometer at the hip using an elastic waist strap. Children were instructed to wear the accelerometer during waking hours, except when bathing or swimming. Physical activity intensities were categorized as sedentary, light, moderate, and vigorous physical activity using validated cut-points (sedentary ≤ 100 counts per minute; light 100 to 2295 counts per minute; moderate 2296 to 4012 counts per minute; vigorous ≥ 4012 counts per minute)^30,31^. Participants were required to have a minimum of 10 h of valid accelerometry data across at least four days in the seven-day measurement period to be included in the analysis.

#### Muscle morphology

We elected to measure the morphology of the multifidus muscles at the lower lumbar spine owing to their key role in spinal stabilization^32,33^. The multifidus is the largest muscle to cross the lumbosacral joint^34^ and contributes two-thirds of lower lumbar stabilization^34^, the spinal region with the highest prevalence of degenerative changes^35^. These characteristics have generated significant clinical interest as spinal instability is a theorized mechanism of back pain used to justify therapies from exercise to surgical fusion^36–38^. Increased stiffness of the lumbar multifidus is associated with low back pain^39^, and a recent systematic review identified several studies reporting associations between low back pain and morphologic changes to the multifidus muscles, including fatty infiltration^10^. Clinically, lumbar multifidus morphology may have prognostic relevance^40^ and appears to be modifiable with exercise training^41,42^.

The proportion of IMAT in the lower lumbar multifidus muscle was examined using open, low-field 0.2 T MRI (Seimens, AG, Erlangen, Germany) and a body spine surface coil. Axial images were positioned by a plane perpendicular to the median of the T2 sagittal lumbar image^43^, perpendicular to the posterior surface of the back muscles of interest and tangential to the posterior corner of the upper vertebral body. These images were obtained using T1-weighted spin echo (300/26 repetition time/echo time), with 4 mm slice thickness, 280 mm^2^ field of view, and a 120 × 256 matrix. Additional details have been previously reported^11,14,44^.

We used sliceOmatic software [TomoVision, Magog, Canada] to identify total cross-sectional area (CSA), muscle (i.e., fat-free) CSA, and IMAT CSA in the lumbar multifidus muscles at the L4 and L5 (4^th^ and 5^th^ lumbar) spinal levels bilaterally. This software, which is reliable for MRI fat assessment^45^, has been used extensively to quantify adipose and muscle tissue in various regions, including the lumbar multifidus^46,47^.

We applied a histographic thresholding procedure to identify a threshold value distinguishing adipose tissue and muscle tissue. This method accounts for numerous variables within images, including image intensity based on bodily location and the degradation of muscle to fat that may occur gradually and is described in greater detail elsewhere^48^. We calculated the maximum proportion of IMAT across the four muscle regions and classified them according to 1) low-, mid-, and high-fat tertiles and 2) their patterns of change over time (persistent, increasing, decreasing). For example, a child ranked in the lowest tertile at time one and time two would be categorised as following a ‘persistent-low’ pattern of change. A child ranked at a higher or lower tertile at time two (relative to time one) would be categorised as following an “increasing” or “decreasing” pattern of change, respectively.

#### Low back pain outcomes

Low back pain data were collected via interviews^49^ modeled after a questionnaire previously used to measure spinal pain as part of a national survey of children^4^. At ages 16 and 21 years, participants reported the occurrence of impactful low back pain in the preceding year when the pain resulted in one or more of the consequences: interference with sports, play, or school attendance, or if the pain required evaluation or treatment from a healthcare provider.

### Data analysis

All analyses were performed using STATA 16.1 (StataCorp, College Station, Texas, USA). We calculated tertiles (low, mid, high) of lumbar multifidus IMAT and categorized patterns of change in tertile groupings from age 13 to 16 years as decreasing, increasing, persistent low, persistent mid, or persistent high.

We constructed separate logistic regression models to investigate the associations between minutes spent in each physical activity intensity category at age 16 and low back pain at 21 (objective 1). Similar logistic regression models were constructed to examine the associations between the patterns of change in lumbar multifidus IMAT from age 13 to 16 years and low back pain at age 21 (objective 2). We also modeled the associations between physical activity at age 9 or 16 years and patterns of change in lumbar multifidus IMAT from age 13 to 16 using multinomial regression (objective 3). For all models that included physical activity, we included accelerometer wear time as a covariate to account for interindividual differences in measurement time. To improve the interpretability of physical activity model outcomes, parameter estimates were expressed as the change in the outcome associated with a 10-min change in activity. Evidence from prior studies shows sex and BMI to be potential causes of physical activity^27^, lumbar multifidus muscle morphology^11^, and low back pain^3,4,11,50,51^. Therefore, we additionally controlled for sex and BMI as potential confounders in fully adjusted models. We investigated the need to account for clustering by introducing a school identifier as a random effect in the model. There were no apparent clustering effects as indicated by a non-significant likelihood ratio test comparing the goodness of fit between the mixed- and fixed-effects models and trivial differences between the resulting parameter estimates. Given the added complexity of including random effects, we reported the results of the more parsimonious models.

Model results were reported with odds ratios (OR) for logistic regressions and relative risk ratios (RRR) for multinomial regressions. Models evaluating lumbar multifidus IMAT as an exposure or outcome used the ‘persistent low’ pattern as the reference category. All regression models employed robust standard errors. The level of significance was 0.05 for all analyses.

### Ethical approval

Ethical approval was granted by The Regional Committees on Health Research Ethics for Southern Denmark (reference nos. 20000045, 96/272), and the University of New Brunswick (reference no. REB#2019–056). Use of the data was approved by the Danish Data Protection Agency (reference no. 2000-5-3-0037).

## Data Availability

Due to legislation in Denmark, data are not publicly available, but can be shared with researchers on reasonable request.
